# Off-label use of interwoven carotid stent in common femoral artery occlusion after surgery

**DOI:** 10.1016/j.jvscit.2023.101214

**Published:** 2023-05-16

**Authors:** Luca Ferretto, Matilde Zamboni, Matteo Vincenzi, Riccardo Bozza, Sabrina Scian, Sandro Irsara

**Affiliations:** a Vascular and Endovascular Surgery Section, Vascular Surgery and Angiology Unit, AULSS 1 Dolomiti, San Martino Hospital, Belluno, Italy; b Department of Radiology, AULSS 1 Dolomiti, San Martino Hospital, Belluno, Italy

**Keywords:** Carotid stent, Chronic limb-threatening ischemia, Common femoral artery, Endovascular, Off-label use

## Abstract

Open surgery is the gold standard for treating common and deep femoral arterial lesions. Nevertheless, significant data have emerged in recent years supporting an endovascular strategy for this peculiar anatomic region, despite certain disadvantages, including the requirement for strong compression resistance and excellent flexibility and conformability when stents are implanted. We present a case of critical limb ischemia due to total common and deep femoral arteries occlusion after endarterectomy that resulted in a very tapered lesion. It was successfully treated with percutaneous angioplasty and off-label application of an interwoven nitinol Roadsaver carotid artery stent, which demonstrated good adaptability.

Open surgery is considered the gold standard for treatment of common femoral artery (CFA) and deep femoral artery (DFA) atherosclerotic lesions, although the role of endovascular (EV) techniques is debated.^1^, ^2^, ^3^ We present a case of critical limb ischemia (CLI) due to total occlusion of the CFA and proximal DFA previously treated with surgical endarterectomy (EA), successfully managed with a total EV approach. The patient provided written informed consent for the report of her case details and imaging studies.

## Case report

An 83-year-old woman presented to our department complaining of rest pain and gangrene of the first toe of the right foot. Six months before, she had undergone EA of the right CFA and DFA for CLI, Rutherford class 4. Computed tomography angiography revealed occlusion of the CFA and DFA. The superficial femoral artery (SFA) was patent at the middle to distal part. The patient was obese (body mass index, 35 kg/m^2^) and had diabetes, chronic heart failure (ejection fraction, 37%), and atrial fibrillation. After the previous EA, she developed severe, infected dehiscence of the surgical wound, which was treated with specific antibiotics, repeated surgical debridement, and application of negative pressure therapy. Our clinical examination revealed no active signs of infection but extensive scar tissue was present surrounding the right CFA, detected by physical examination and confirmed by computed tomography angiography.

**Fig 1:**
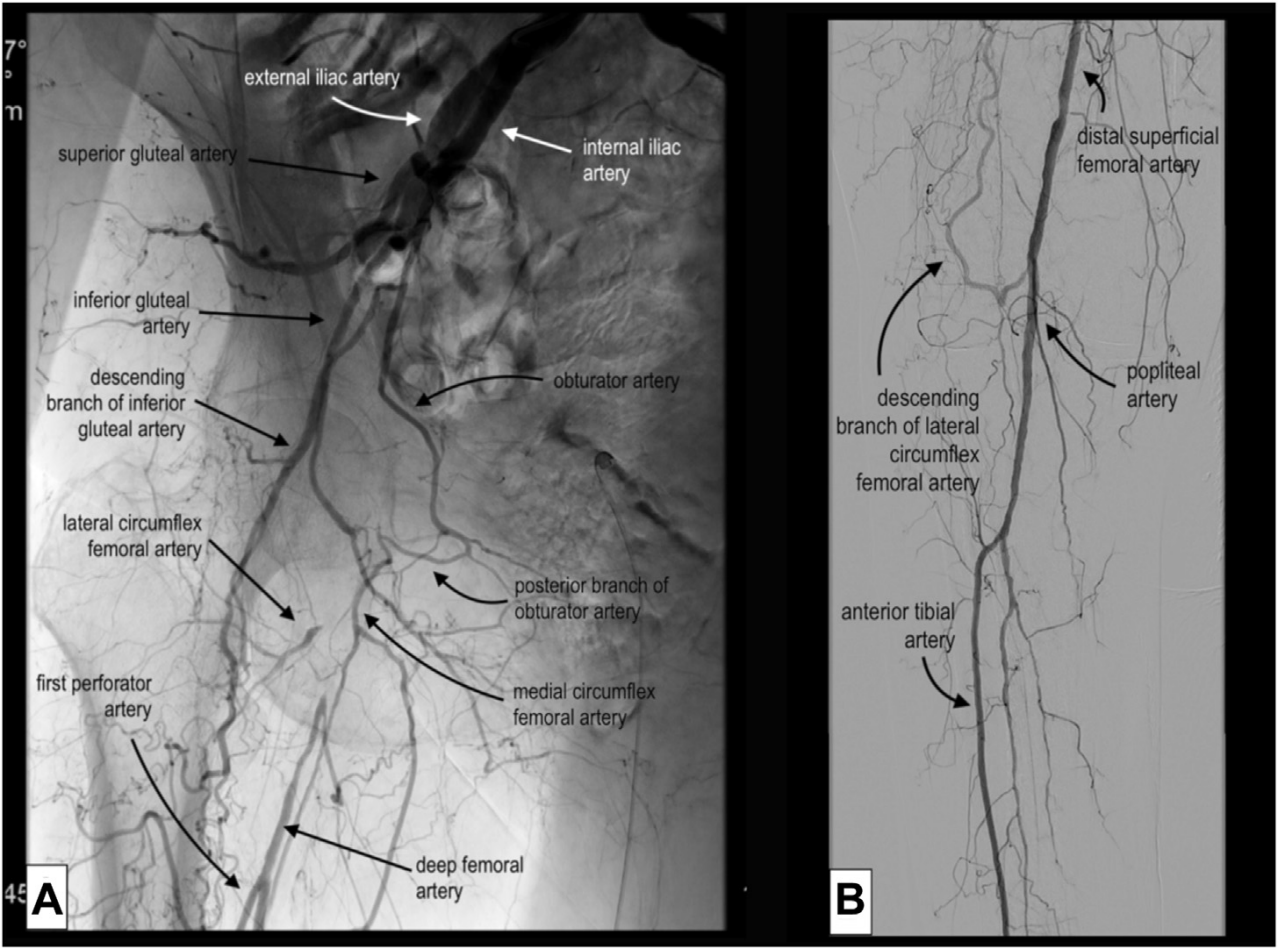
**A,** Preoperative digital subtraction angiogram showing total occlusion of the right common femoral artery (CFA) and first third of the deep femoral artery (DFA). The external iliac artery is not visible in this frame but was patent until almost the origin of the epigastric artery. **B,** The superficial femoral artery (SFA) was occluded during previous endarterectomy (EA) and was supplied in the middle to distal third by collateral vessels. Runoff to the leg was maintained by the distal SFA (supplied by collateral vessels from the DFA), popliteal artery, and anterior tibial artery.

**Fig 2:**
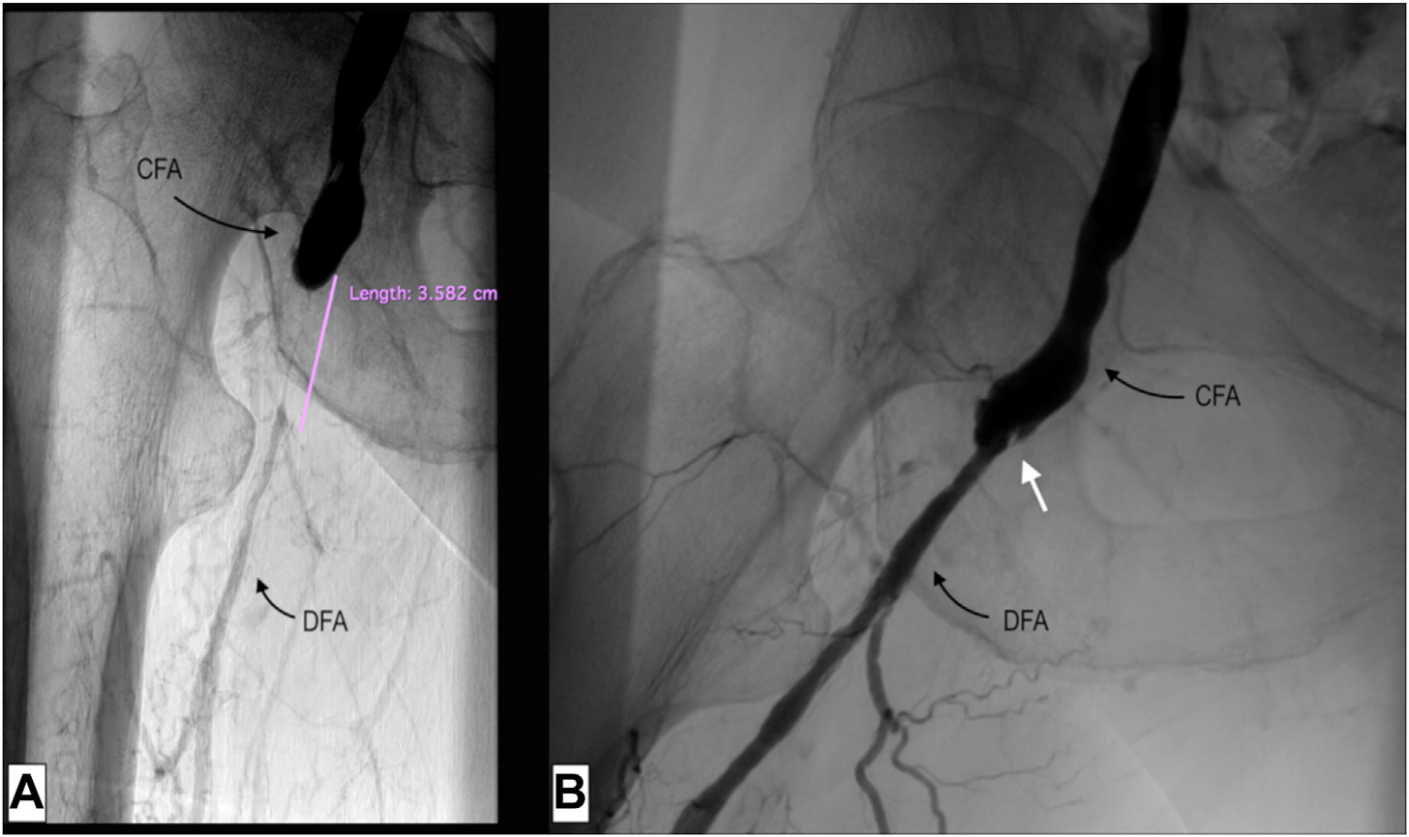
**A,** The lesion length appeared to be ∼36 mm after crossing the occlusion, shorter than previously estimated. Crossing was obtained by combined maneuvers with a diagnostic 5F catheter (MPA; Cordis Corp), a supportive 2.6F microcatheter (CXI; Cook Medical Inc), and a 0.018-in. guidewire (V18; Boston Scientific). **B,** After multiple dilatations with plain old balloon angioplasty and drug-eluting balloons, a short dissection was noted at the distal part of the common femoral artery (CFA; *white arrow*). The image was taken at hip flexion of ∼45° and revealed no bending of the deep femoral artery (DFA).

**Fig 3:**
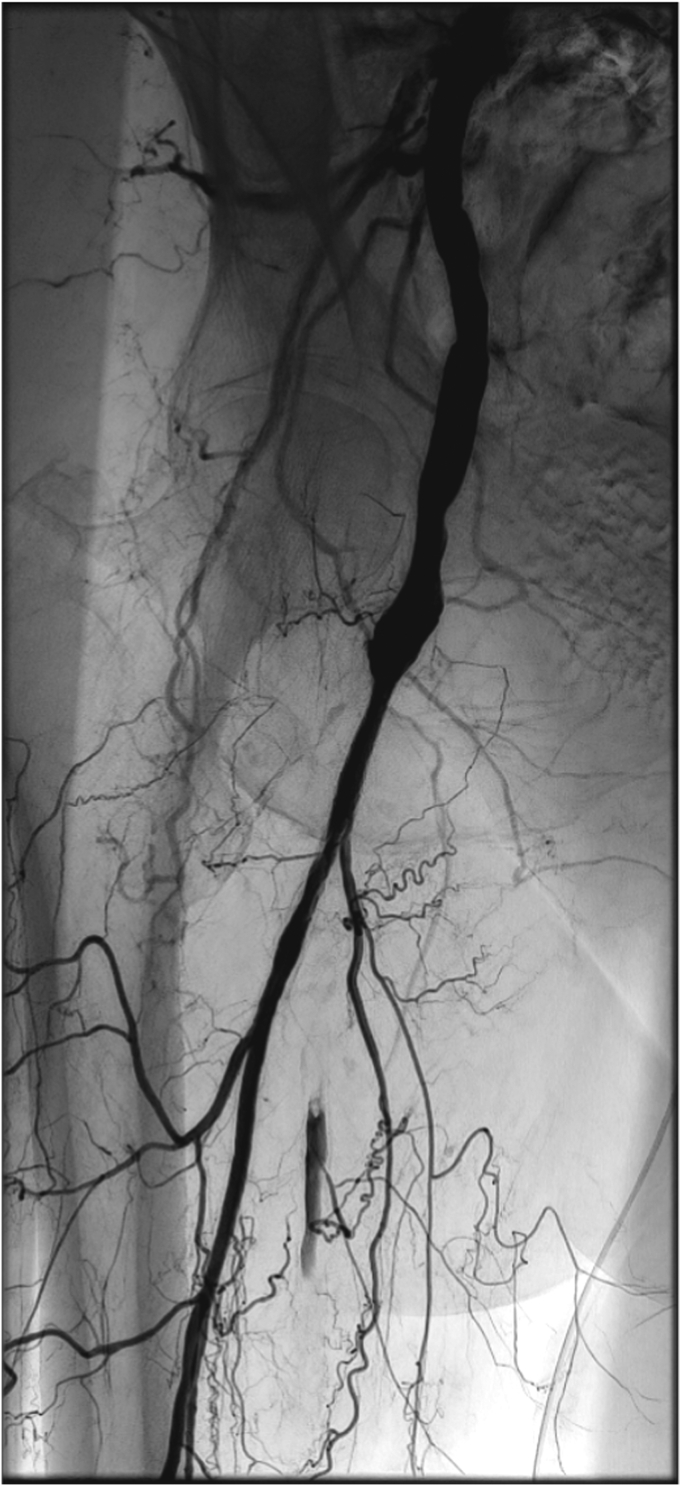
Completion digital subtraction angiogram demonstrating complete recanalization of the common femoral artery (CFA) and deep femoral artery (DFA) and good patency of the distal DFA and its branches.

**Fig 4:**
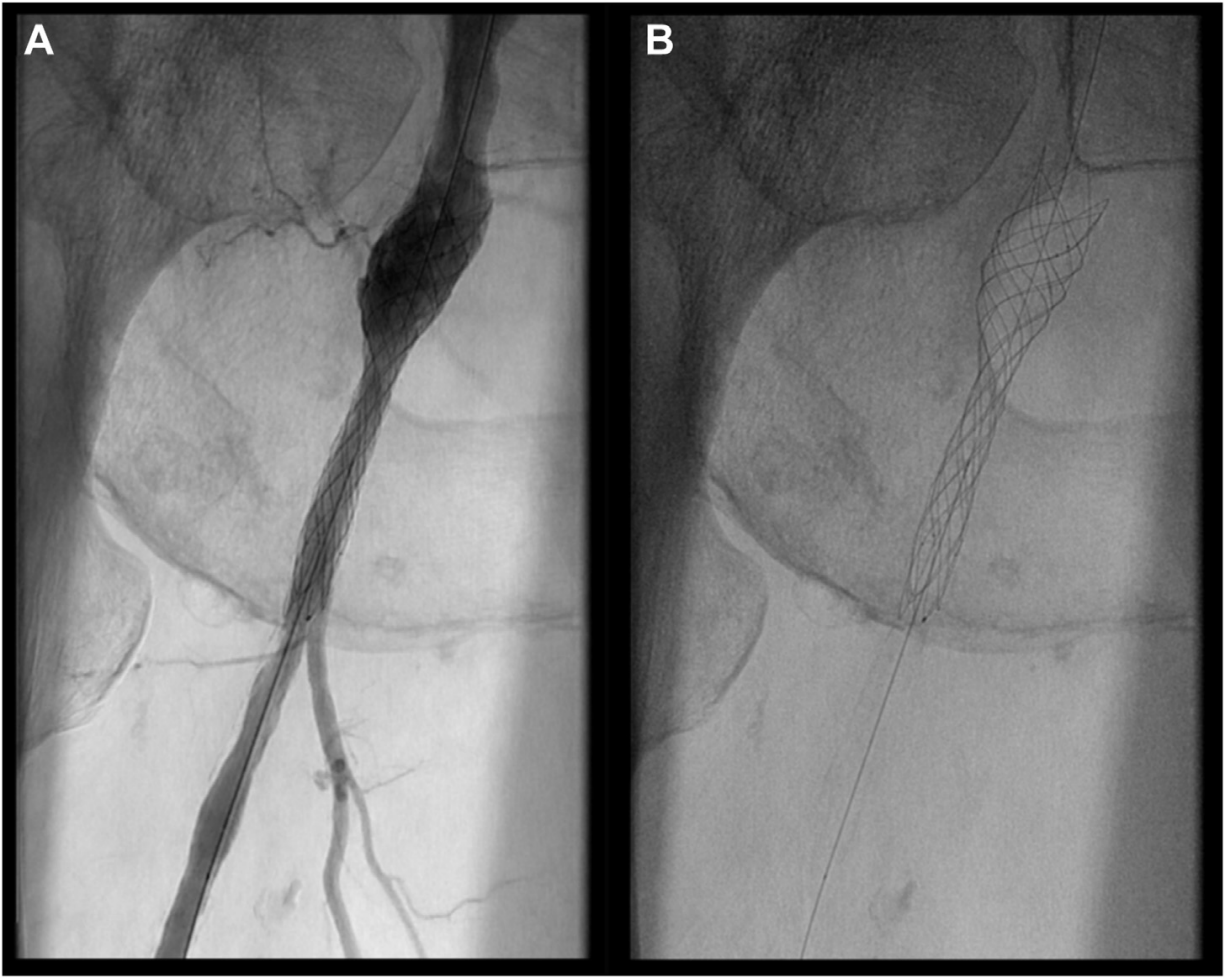
**A,** Detail of completion digital subtraction angiography focusing on the Roadsaver stent after deployment and postdilatation on the deep femoral artery (DFA). The stent adapted correctly to the lesion, with good apposition in both the common femoral artery (CFA) and the DFA, despite the large difference in diameters between the two arteries. **B,** The interwoven nitinol design permits superior adaptability to the tapered morphology of the lesion, with maintenance of the correct structure of the entire stent. The angles between the wires are not very dissimilar in the DFA (stent elongated) vs in the CFA (stent at nominal diameter), which are crucial for maintaining good flexibility of the device.

**Fig 5:**
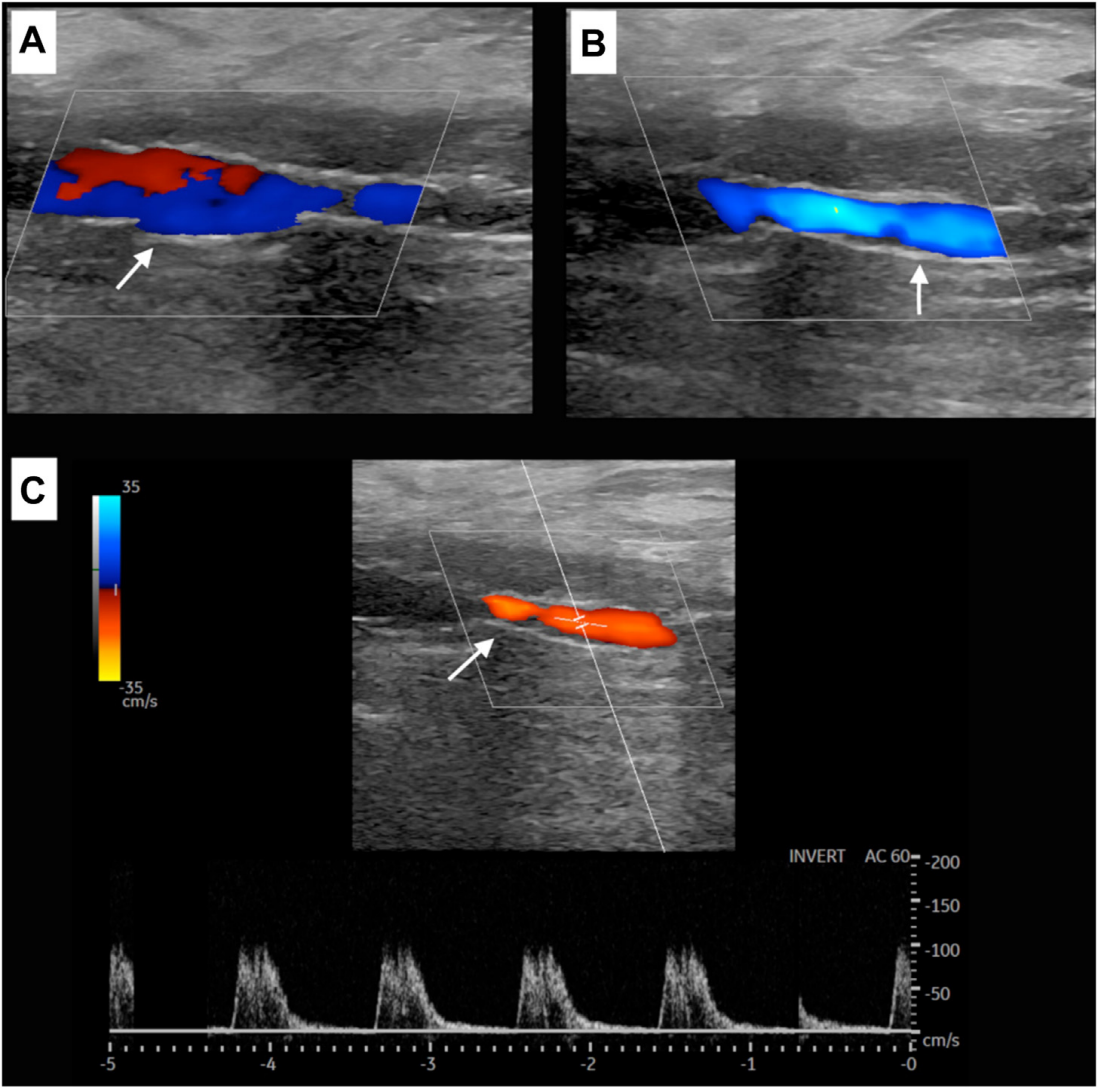
At 6 months after intervention, color Doppler ultrasound scans demonstrating regular stent patency (*white arrow*) in common femoral artery (CFA; **A**) and deep femoral artery (DFA; **B**), with no signs of stent fracture or intraluminal hyperplasia. The peak systolic velocity flow within the stent was ∼100 cm/s (**C**).

Considering the hostile anatomy of the groin, we planned a total EV approach to the right CFA and DFA. A baseline angiogram confirmed the preoperative findings (Fig 1). Intraluminal crossing of the total occlusion of the right CFA and DFA was obtained (Fig 2, *A*) and dilatation with plain old balloons (Sterling, 3 × 40 mm and 5 × 40 mm; Boston Scientific) performed. Subsequently, a focal residual and resistant stenosis was dilated with a noncompliant 5 × 20-mm Dorado balloon (BD). Next, a drug-eluting balloon (In.Pact Admiral, 6 × 60 mm; Medtronic) was maintained inflated at a nominal pressure for 3 minutes. After angioplasty, a short dissection occurred (Fig 2, *B*). Thus, we decided to stent the lesion. A 10 × 20-mm braided nitinol carotid stent (Roadsaver; Terumo Corp) was deployed, postdilated at 5 mm in the DFA, with good adaptability to the tapered vessel (∼5 and ∼ 10 mm in the DFA and CFA, respectively; Figs 3 and 4). Three days after the procedure, the patient underwent amputation of the first and second toes of the right foot. The rest pain had resolved after 2 days, and 3 months later, she had completely recovered. Transcutaneous oximetry was 52 mm Hg at 1 month and 60 mm Hg at 6 months. Duplex ultrasound scanning at 6 months after revascularization showed no restenosis, with no narrowing or flow increase observed. The peak systolic velocity within the stent was ∼100 cm/s (Fig 5).

## Discussion

The DFA is the main vessel providing arterial blood to the thigh muscles and femur and represents the main collateral pathway to maintain distal extremity perfusion in the case of occlusion of the SFA. Thus, restoring DFA patency will often be enough to avoid CLI. Collateralization of the SFA by the DFA is rarely developed enough to permit healing of a lesion, although it occurred in our patient (Fig 1), for whom a normal transcutaneous oximetry value was measured at 1 and 6 months after the procedure.

For our patient, the ideal surgical intervention to restore inline flow to the foot would have been bypass from the right external iliac artery to the ipsilateral SFA. Axillary-to-DFA, axillary-to-SFA, and obturator-to-SFA bypass was also considered as an extra-anatomic solution to avoid passage into the groin. However, considering the good collateralization of the SFA, we opted for EV recanalization, reserving open surgical intervention as a second choice.

During recanalization, we were concerned about the risk of embolization to the DFA. However, we judged it acceptable, believing the occlusion was mainly due to neointimal hyperplasia and not thrombus. After endoluminal angioplasty, with plain old balloon angioplasty and drug-eluting balloons, a good lumen was obtained. Even if, in this case, there was not a risk of plaque protrusion, as often happens in native CFA stenosis, we preferred to stent the lesion because of the presence of a short dissection (Fig 2, *B*). If no dissection had occurred after angioplasty, we would have been in doubt regarding whether to perform stent placement. Stenting of the CFA or DFA is questioned because of the high mobility of the anatomic area and the high compressive forces that could lead to stent fracture. However, evidence of the safety of EV techniques has been increasing, starting with the TECCO (endovascular versus open repair of the common femoral artery) trial,^2^ which demonstrated comparable results for EV treatment vs EA. More recently, routine stenting of CFA lesions appeared to provide better patency and target lesion revascularization rates compared with bailout stenting at short-term follow-up (1-2 years).^3^ Interwoven stents are often indicated as the best tool to stent the CFA and DFA because they have a low chronic outward force but high resistance to compression, which seems to be essential to avoid fractures when deployed under or close to the inguinal ligament. Promising results are coming from implantation of the Supera stent (Abbott Vascular), a helical interwoven nitinol self-expandable stent.^4^ This device has been studied in the femoropopliteal artery.^5^^,^^6^ Nevertheless, what concerned us more was the extremely tapered morphology of the lesion. In our experience to date, the Supera stent is unable to adapt very well in such different diameters. Additionally, its largest available diameter on the market is 7.5 mm, insufficient for the proximal CFA diameter of our patient.

While analyzing the structural features of the Supera stent, we explored the use a similar device we are familiar with: the Roadsaver carotid artery stent. The Roadsaver stent is a dual layer stent with an outer interwoven nitinol stent and an inner micromesh layer with very small pore sizes. The Roadsaver stent was shown to have very good adaptability in tapered anatomy, according to our experience derived from carotid artery stenting. We opted for a Roadsaver 10 × 20-mm stent, where 20 mm is the length of the inner layer and the overall length of the device is 35 mm. After complete deployment, the total length of the stent was ∼60 mm owing to the elongation into a 5-mm DFA. When a Roadsaver stent is released into a small vessel, the nitinol wires tend to maintain the structure with a nominal diameter, despite elongation or bending, as shown in Fig 4, *B*, where the angles between the wires are not very dissimilar in the DFA (stent elongated) or the CFA (stent at nominal diameter). An analysis of the biomechanical properties of this stent type showed that the angle between the wires is essential for maintaining its flexibility.^7^ Moreover, compared with other interwoven stents in an experimental model of a stenotic rigid vessel, the Roadsaver stent demonstrated a lower reaction force on the vessel wall at the point of maximum stenosis.^8^ This could lead to a minor propensity to neointimal hyperplasia formation.

The Roadsaver stent was created for application in the carotid artery and its use in the CFA and DFA is off label. However, we believe that the structure of the external nitinol layer, very similar to that of the Supera stent, could represent a key feature of this tool for use in the CFA. Finally, the internal micromesh layer ensures high coverage of the treated artery, potentially leading to a reduction in restenosis, which has been observed in other sites with covered stents,^9^^,^^10^ but has not yet been tested on CFA and DFA with this device.

## Conclusions

In the present report, we suggest the off-label application of the Roadsaver carotid artery stent in EV treatment of CFA and DFA occlusion. New studies are needed to determine whether a real advantage exist for using this device for CFA and DFA lesions, and a brand-new tool could be developed to adapt even better to the unique area of the CFA.
